# Supplementary material for: Inhibition of TRPV1 channels by a naturally occurring omega-9 fatty acid reduces pain and itch
Source: Nat Commun. 2016 Oct 10;7:13092. doi: 10.1038/ncomms13092 (PMC5062500; doi:10.1038/ncomms13092)
Supplement: Supplementary Information — Supplementary Figures 1-7 and Supplementary Tables 1-3 [file ncomms13092-s1.pdf]

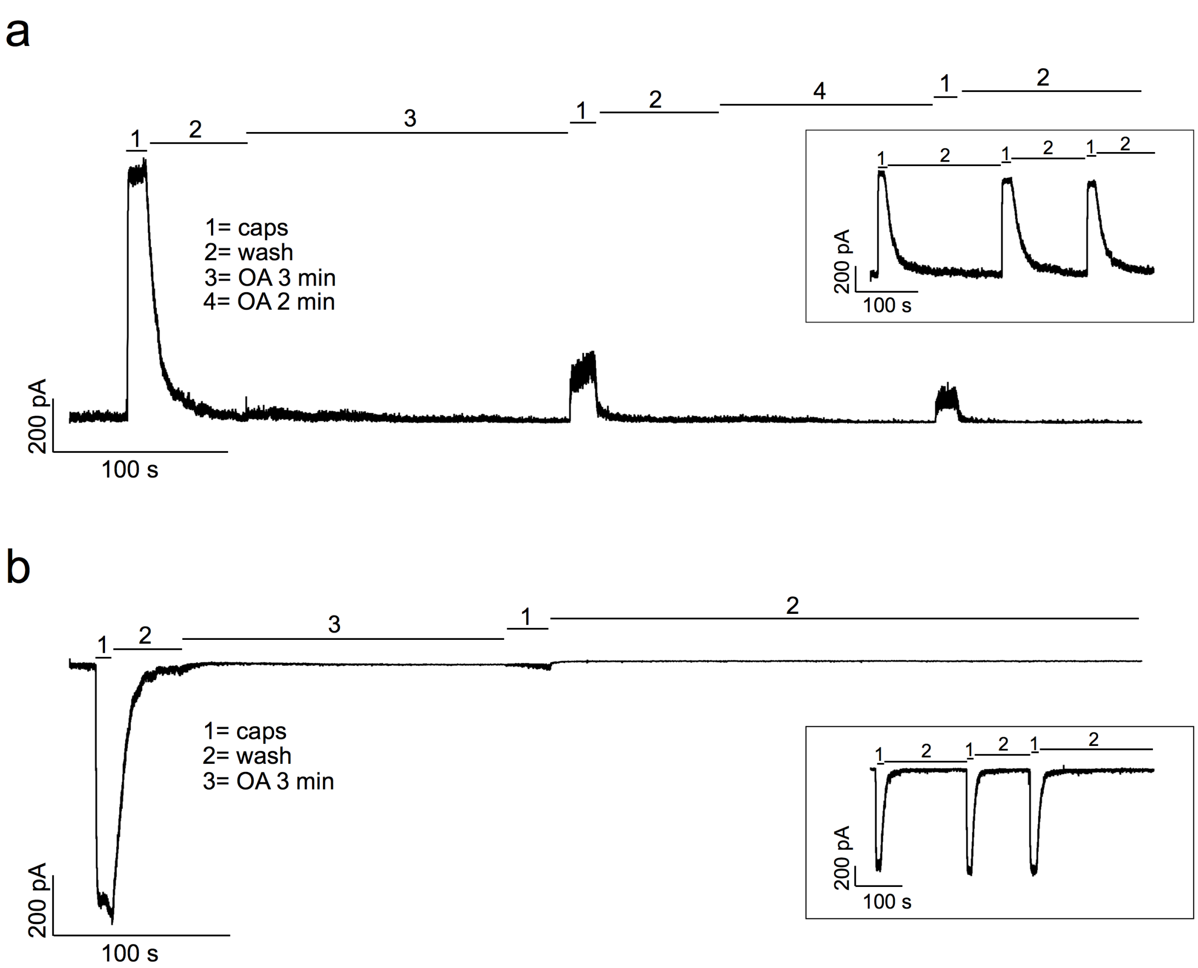

Supplementary Figure 1. Oleic acid does not activate rTRPV1 currents

(a) Representative capsaicin evoked TRPV1 currents obtained by holding the membrane voltage at +40 mV and at (b) -40 mV. An absence of current activation by 5 μM OA alone is shown by bars 3 and 4 in (a) and by bar 3 in (b) (n=6 for each voltage). The insets in both (a) and (b) show that the TRPV1 channel does not desensitize in response to repeated capsaicin applications (bar 1), in experiments at both voltages (+40 and -40 mV, n = 3 for each case).

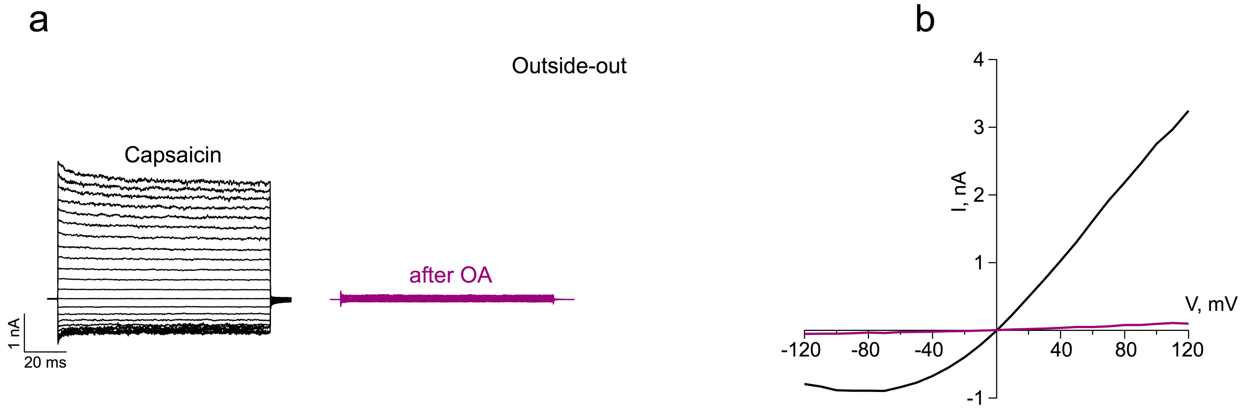

Supplementary Figure 2. Inhibition of capsaicin-activated currents by oleic acid

(a) Traces shown are representative of 10 experiments. Currents from outside-out patches were obtained by stepping the voltage in 10 mV increments from 0 mV to +/-120 mV for 100 ms. Patches were first exposed to 4 μM capsaicin, washed, and then to 5 μM oleic acid (OA) for 5 min (not shown) before re-exposing to capsaicin (magenta traces). (b) Current-voltage relationships (for currents in a) in the presence of 4 μM capsaicin (black) and of capsaicin after 5 min of 5 μM OA exposure (magenta).

a

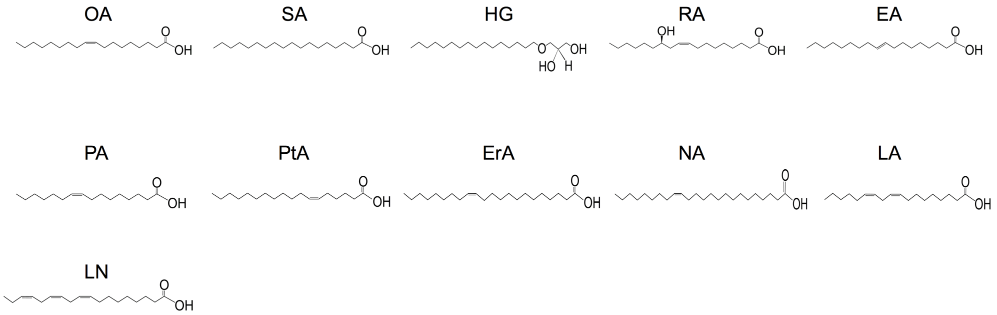

b

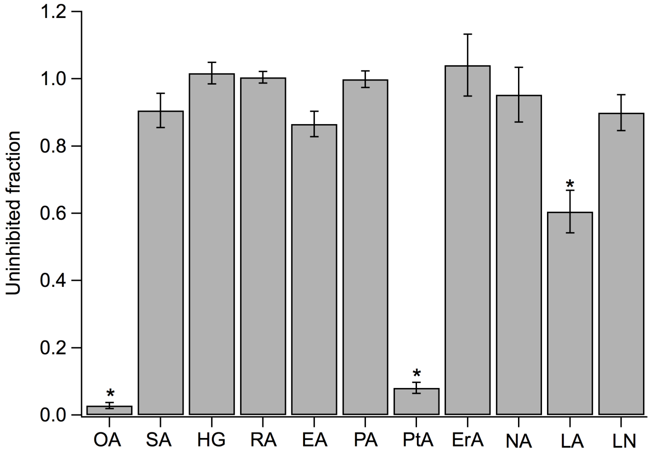

Supplementary Figure 3. Specificity of inhibition of TRPV1 by fatty acids

(a) Chemical structures of cis-9-Octadecenoic acid or oleic acid (OA); 1-Heptadecanecarboxylic acid or stearic acid (SA); 1-C16 ether MG 1-O-hexadecyl-*sn*-glycerol or hexaglycerol (HG); (*R*)-12-Hydroxy-*cis*-9-octadecenoic acid or ricinoleic acid (RA); 9-octadecenoic acid or elaidic acid (EA); (9Z)-hexadec-9-enoic acid or palmitoleic acid (PA); 6-octadecylenic acid or petroselinic acid (PtA); cis-13-docosenoic acid or erucic acid (ErA); cis-15-tetracosenoic acid or nervonic acid (NA); cis-9, cis-12-octadecadienoic acid or linoleic acid (LA) and cis,cis,cis-9,12,15-octadecatrienoic acid or linolenic acid (LN). (b) Normalized remaining currents after application of the fatty acids indicated in the abscissa obtained by dividing the remaining capsaicin-activated currents after 5 μM OA by the initial capsaicin-activated currents. OA, PtA and linoleic acid significantly inhibited capsaicin-activated currents arising from TRPV1 channels. The data was obtained at +120 mV and normalized to activation by 4 μM capsaicin (n= 7-27). * denotes $p< 0.0001$.

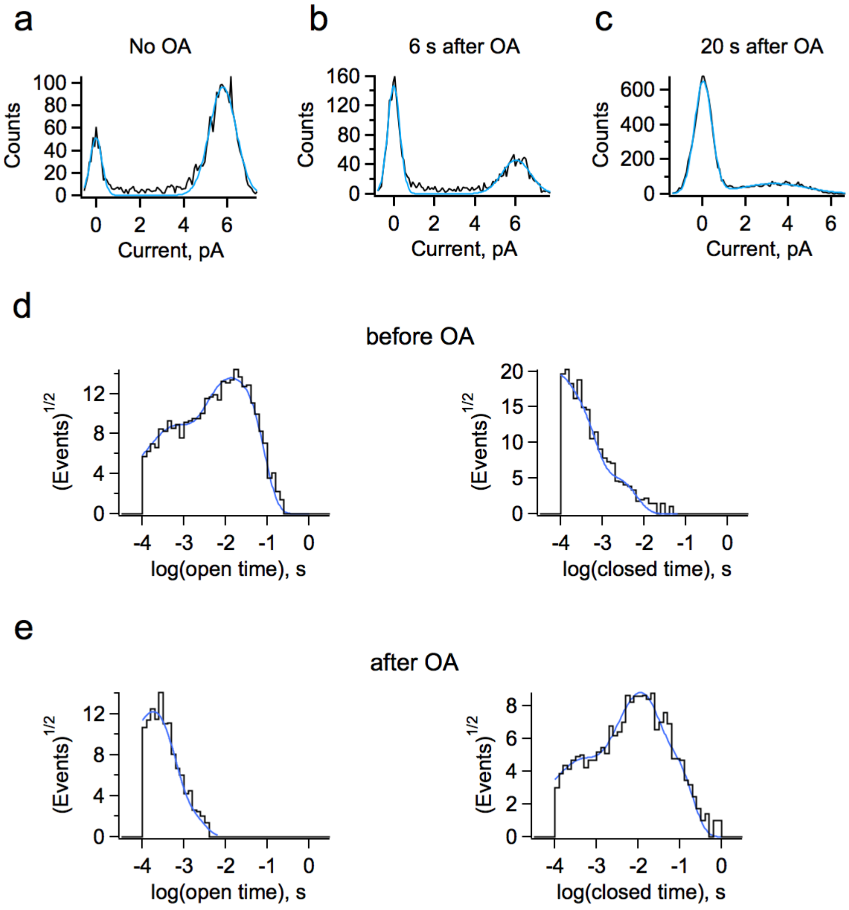

Supplementary Figure 4. Effects of oleic acid on the kinetics of TRPV1 single channel currents (a) Single channel current behavior of TRPV1 in the absence of OA. All-points histogram of a 200 ms current sweep in the absence of OA. The histogram peak at 0 pA is a closed state level, while the open state level current is indicated by the peak on the right, with a mean amplitude of 5.79 ± 0.86 pA at 120 mV. (b) All-points histogram 6 seconds after OA application, the current amplitude is 6.02 ± 0.99 pA. (c) Histogram 20 seconds after OA application, current amplitude is 3.35 ± 2.14 pA. (d and e) Kinetic analysis of channel gating before and after exposure to OA. Open and closed dwell time histograms are plotted in the left and right columns, respectively. Data in the presence of capsaicin (d) and the last 15 seconds of the OA inhibition. (e). Intermediate times were not analyzed because of the non-stationarity of the data. Open and closed time histograms where fitted to the sum of three exponentials, except for the open time histogram after OA, which was fit to two exponentials. The value of the time constant of each of the components and its amplitude are given in Supplementary Table I.

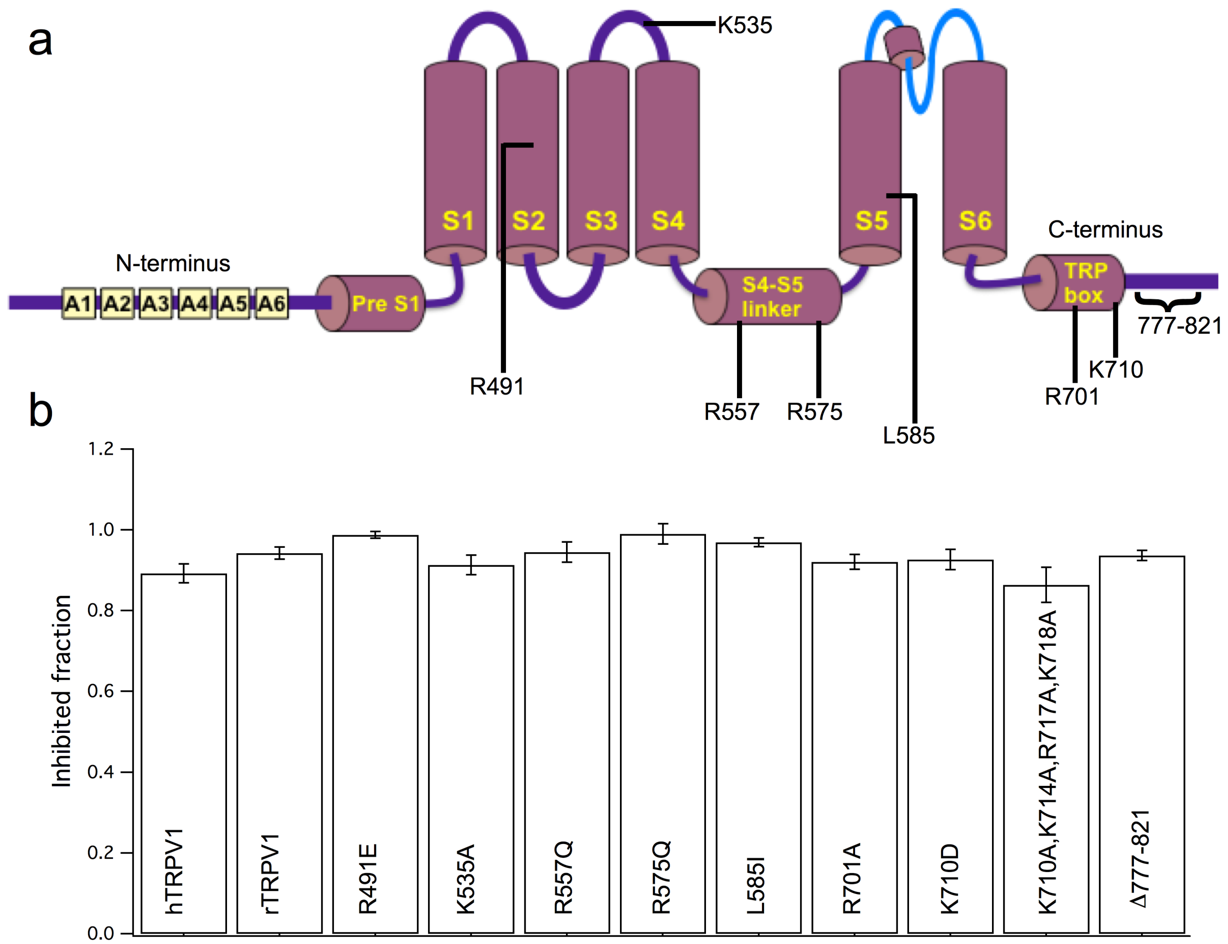

Supplementary Figure 5. Effects of oleic acid on wild type and mutant rTRPV1 channels

(a) Schematic representation of a TRPV1 subunit. Transmembrane regions (S1-S6) are indicated in dark pink, the N- and C-termini as well as linker loops are represented by purple lines, A1-A6 are ankyrin repeats, and the light blue line represents the pore region. Mutations in different regions are indicated in black. (b) Fraction of capsaicin-inhibited currents after application of 5 μM OA in wild-type channels (human and rat TRPV1, hTRPV1 and rTRPV1, respectively) and in mutant rTRPV1 channels. The data was obtained at +120 mV and normalized to activation by 4 μM capsaicin (n=5-11).

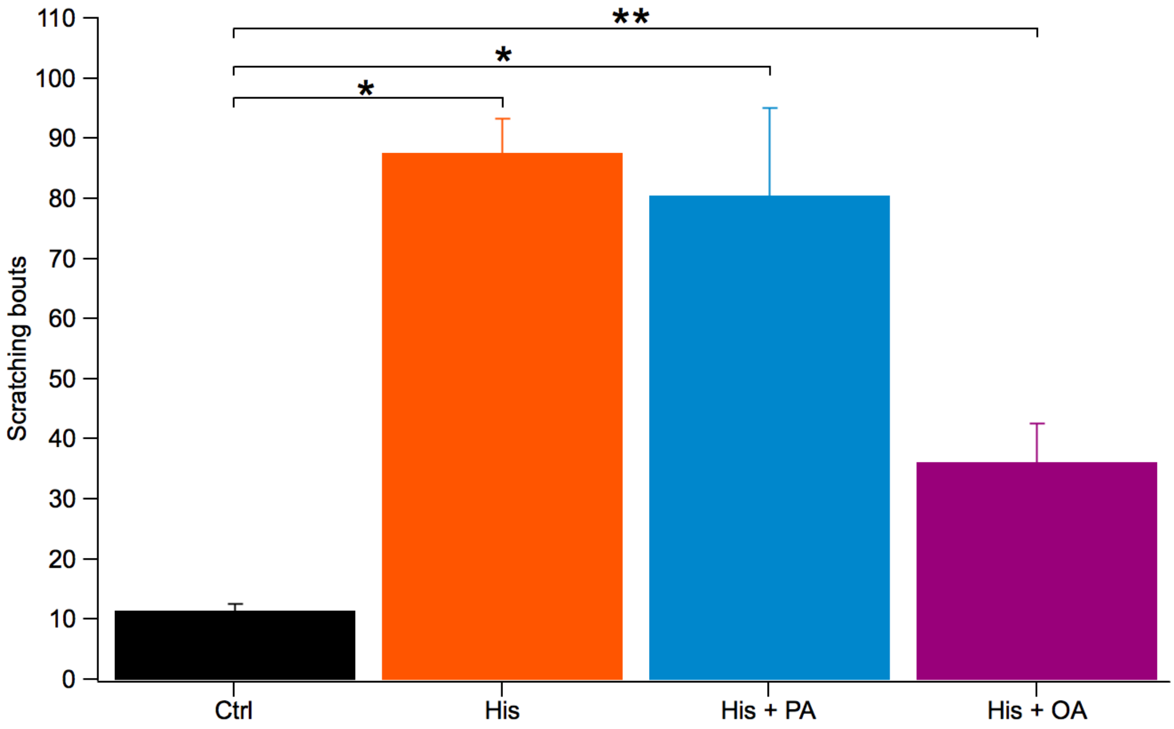

Supplementary Figure 6. Histamine-induced itch is partially inhibited by oleic acid

The average number of scratching bouts were: 11.6 ± 1.1 for vehicle-injected (DMEM + 1% BSA; Ctrl; n = 10); 87.7 ± 5.5 for histamine (50 µg of His; n= 13); 80.6 ± 15 for 50 µg histamine + palmitoleic acid (1.25 µg/g of weight of PA) (n= 8) and 36.1 ± 6.3 for 50 µg histamine + OA (1.25 µg/g of weight) (n= 11). *denotes $p < 0.0001$ and ** is $p < 0.001$ as compared between groups by brackets.

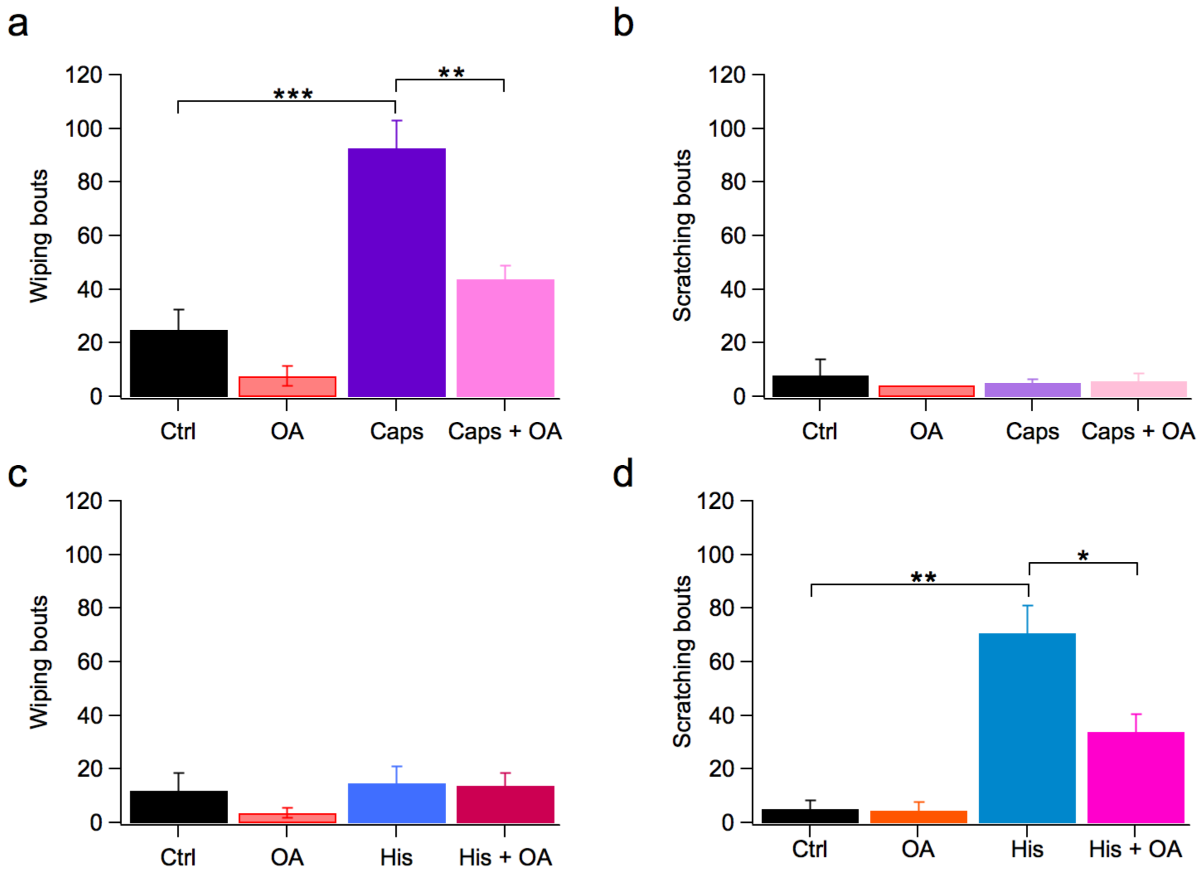

Supplementary Figure 7. Inhibition of pain and itch in the "cheek" model

(a) Capsaicin-induced pain behavior. The average number of wiping bouts were: 25.2 ± 7.6 for vehicle-injected (Ctrl; n = 5); 7.6 ± 3.7 for OA (5 µg; n = 5); 92.7 ± 10.3 for capsaicin (0.1 µg, Caps; n = 9) and 43.9 ± 4.8 for capsaicin + OA (n = 9). (b) Capsaicin-induced itch behavior. The number of scratching bouts were: 8± 5.7 for vehicle-injected (n = 5); 4.2 ± 2 for OA (5 µg; n = 5); 5.3 ± 1.1 for capsaicin (0.1 µg; n = 9) and 6 ± 2.9 for capsaicin + OA (n = 9). (c) Histamine-induced pain behavior. The average number of wiping bouts were: 12 ± 6.4 for vehicle-injected (n = 5); 3.8 ± 2 for OA (10 µg; n = 5); 15 ± 6.3 for histamine (His; 30 µg; n = 6) and 13.9 ± 4.7 for histamine + OA (n = 8). (d) Histamine-induced itch behavior. The average number of scratching bouts were: 5.2 ± 3 for vehicle-injected (n = 5); 4.6 ± 3 for OA (10 µg; n = 5); 70.8 ± 10.2 for histamine (His; 30 µg; n = 6) and 34 ± 6.4 for histamine + OA (n = 8). * denotes $p < 0.01$, ** is $p < 0.001$ and ***$p < 0.0001$, as compared between groups by brackets.

Supplementary Table 1. Parameters of the fit of the distributions of dwell times to three exponentials

Histogram	Amplitude 1	Time constant 1 (ms)	Amplitude 2	Time constant 2 (ms)	Amplitude 3	Time constant 3 (ms)
Closed time in capsaicin	12.9	0.03	21.8	0.17	8.3	1.77
Open time in capsaicin	7.1	0.27	6.1	4.14	20.1	23.29
Closed time after OA	3.7	0.20	9.2	7.49	8.4	52.7
Open time after OA	16.4	0.15	-	-	5.2	0.69

Supplementary Table 2. Effects of oleic acid (OA) on capsaicin- (Caps) and lysophosphatic acid (LPA)- induced pain

	Pain (Paw-licking time, s)
Ctrl (0.3% ethanol)	16.7 ± 2.9
Oleic Acid	15.0 ± 1.8
Caps	39.4 ± 2.1*
Caps + OA	24.4 ± 2**
Ctrl (DMEM +1%BSA)	12.1 ± 1.9
LPA	$43 \pm 1.6^{+}$
LPA + OA	$27.2 \pm 2.9^{++}$

*p< 0.001 for Caps vs Ctrl; **p< 0.001 for Caps vs Caps + OA; $^{+}$p< 0.0001 for Ctrl vs LPA and ++ p< 0.001 for LPA vs LPA + OA.

Supplementary Table 3. TRPV1-dependent itch inhibition by oleic acid

	Itch (Number of scratching bouts in 20 min)
	**WT mice**
Control (DMEM+1%BSA)	11.6 ± 1.1
Oleic acid (OA)	7.3 ± 1.6
Cyclic phosphatidic acid (cPA)	31.6 ± 2.7*
cPA + OA	17.3 ± 3.2**
	**Trpv1$^{-/-}$ mice**
Control (DMEM+1%BSA)	6.7 ± 1.7
cPA	11.1 ± 2.1^{+}

For WT mice: *$p < 0.0001$ for cPA vs Ctrl; **$p < 0.01$ for cPA vs cPA + OA. $^{+}p < 0.0001$ for WT cPA vs *Trpv1*$^{-/-}$ cPA. See text for details.
